# Transcriptomic Analysis Revealed an Important Role of Peroxisome-Proliferator-Activated Receptor Alpha Signaling in Src Homology Region 2 Domain-Containing Phosphatase-1 Insufficiency Leading to the Development of Renal Ischemia-Reperfusion Injury

**DOI:** 10.3389/fmed.2022.847512

**Published:** 2022-05-10

**Authors:** Sijia Yan, Mingxing Sui, Hongzhe Tian, Jiazhao Fu, Yanfeng Li, Jing Chen, Li Zeng, Xianting Ding

**Affiliations:** ^1^State Key Laboratory of Oncogenes and Related Genes, Institute for Personalized Medicine, School of Biomedical Engineering, Shanghai Jiao Tong University, Shanghai, China; ^2^Department of Organ Transplantation, Changhai Hospital, Navy Medical University, Shanghai, China; ^3^Department of Laboratory and Diagnosis, Changhai Hospital, Navy Medical University, Shanghai, China; ^4^Department of Urology Surgery-General Hospital of Central Theater Command of PLA, Wuhan, China

**Keywords:** kidney transplantation, renal ischemia-reperfusion injury, SHP-1, PPARα signaling, bioinformatics

## Abstract

In kidney transplantation, the donor kidney inevitably undergoes ischemia-reperfusion injury (IRI). It is of great importance to study the pathogenesis of IRI and find effective measures to attenuate acute injury of renal tubules after ischemia-reperfusion. Our previous study found that Src homology region 2 domain-containing phosphatase-1 (SHP-1) insufficiency aggravates renal IRI. In this study, we systematically analyzed differences in the expression profiles of SHP-1 (encoded by *Ptpn6*)-insufficient mice and wild-type mice by RNA-seq. We found that a total of 161 genes showed at least a twofold change, with a false discovery rate <0.05 in Ptpn6 ^+/mev^ mice after IRI and 42 genes showing more than a fourfold change. Of the eight genes encoding proteins with immunoreceptor tyrosine-based inhibitory motifs (ITIMs) that bind to Ptpn6, three were upregulated, and five were downregulated. We found that for the differentially expressed genes (DEGs) with a fold change >2, the most significantly enriched Kyoto Encyclopedia of Genes and Genomes (KEGG) pathways were the cell division pathway and peroxisome-proliferator activated receptor PPARα signaling pathways. Furthermore, the downregulated genes of the PPARα signaling pathway were mainly related to fatty acid absorption and degradation. Using an agonist of the PPARα signaling pathway, fenofibrate, we found that renal IRI was significantly attenuated in Ptpn6 ^+/mev^ mice. In summary, our results show that insufficiency of SHP-1 inhibits the expression of genes in the PPARα signaling pathway, thereby leading to increased reactive oxygen species (ROS) and exacerbating the renal IRI. The PPARα signaling agonist fenofibrate partially attenuates renal IRI induced by SHP-1 insufficiency.

## Introduction

Kidney transplantation is the best alternative therapy for end-stage renal disease. Renal ischemia-reperfusion injury (IRI) refers to a combination of warm ischemic injury, cold preservation injury during procurement and preservation, and injury induced by vascular recanalization during transplantation. IRI is one of the key factors affecting the clinical outcomes of kidney transplantation and remains a major challenge of kidney transplantation (1). After renal IRI, large amounts of reactive oxygen species (ROS) are produced by activated vascular endothelial cells in the kidneys, which can damage cell membranes and mitochondria. Renal tubular epithelial cells (TECs), which form the main group of renal parenchymal cells, are more sensitive than other renal cells to ischemia and hypoxia. IRI can cause excessive inflammatory responses and apoptosis or necrosis of TECs. In addition, the sodium-potassium pumps in TECs become dysfunctional during IRI due to a lack of energy, which directly leads to necrosis and apoptosis of the TECs and exposure of the basement membrane.

Src homology region 2 domain-containing phosphatase-1 (SHP-1), encoded by the *Ptpn6* gene, is a member of the classical phosphatase family that mediates the dephosphorylation of tyrosine. SHP-1 is widely involved in various biological processes, including cell communication through adhesion junctions, signal transduction in the cytokine-receptor pathway, the natural immune response mediated by natural killer cells, and the adaptive immune response of the B/T lymphocyte-receptor signaling pathway (2–4). Previous studies have indicated that SHP-1 can regulate apoptosis, possibly by playing a proapoptotic role (5–7). SHP-1 can also negatively regulate the production of Toll-like receptors mediated proinflammatory factors by inhibiting the activation of the nuclear factor kappa-B and mitogen-activated protein kinase signaling pathways (8). Moreover, SHP-1 can upregulate the production of type I interferon by interacting with IRAK1 (8). It has been reported that ROS produced in the IRI process can inactivate some phosphatases of the protein tyrosine phosphatase (PTP) family via short oxidation, thus fine-tuning tyrosine phosphorylation-dependent signaling pathways (9, 10). Additionally, Krotz et al. found that inhibition of SHP-1 function can increase intracellular ROS concentrations by increasing or prolonging the activation of endogenous ROS release mechanisms (11).

However, the role of SHP-1 in renal IRI has not yet been fully investigated. In a previous study, we demonstrated that SHP-1 was expressed mostly in TECs, not in macrophages, in the kidney cortex. SHP-1 inhibited renal IRI by dephosphorylating ASK1 and suppressing apoptosis of TECs in *Ptpn6* ^+/mev^ mice after renal IRI (12). The goal of this research was to analyze the differentially expressed genes (DEGs) in mice with renal IRI to gain more insights into the potential role of SHP-1 in renal IRI and to determine the underlying mechanisms. Since homozygous mice rarely survive anesthesia and surgery when used as kidney IRI models, heterozygous mice with wild-type littermates by all indicators except SHP-1 expression (decreased SHP-1 expression in heterozygotes) were used in our studies (13).

## Materials and Methods

### Sample and Data Collection

*Ptpn6*^+/mev^ mice maintained on a C57BL/6 background were purchased from Jackson Laboratories. These mice exhibit a T-to-A mutation at a splice consensus site. We designed primers (Supplementary Table 1) containing the mutation site in the middle of the PCR amplicon to genotype wild-type (^+/+^), heterozygous (^+/mev^), and homozygous (^mev/mev^) mice with sequence maps. All animal experiments were undertaken following the National Institutes of Health’s Guide for the Care and Use of Laboratory Animals with the approval of the institutional research ethics committee of Navy Medical University, Shanghai, China.

**FIGURE 1:**
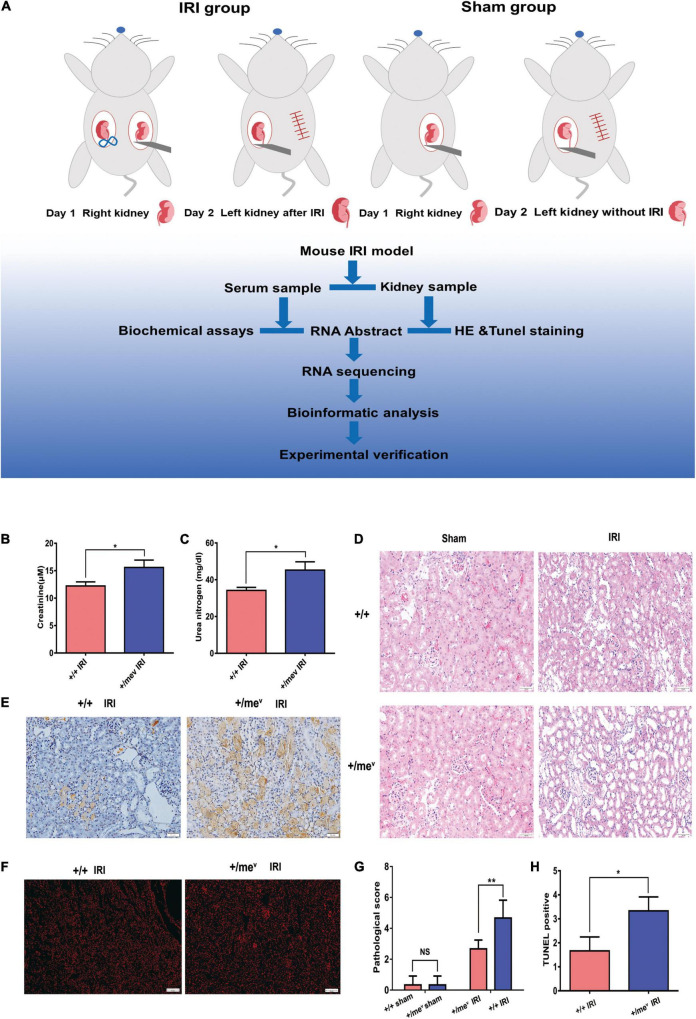
SHP-1 insufficiency *in vivo* aggravated renal IRI and increased apoptosis. **(A)** The steps involved in the establishment of the mouse renal IRI model and analysis process. Briefly, the control group and the heterozygous mice underwent unilateral nephrectomy. After right nephrectomy, the left kidney was clipped for 34 min, and 24 h later, the left kidney was removed for subsequent experiments. **(B)** Creatinine levels of wild-type and *Ptpn6*^+/mev^ mice after renal IRI (*n* = 3 in each group). **(C)** Blood urea nitrogen levels of wild-type and *Ptpn6* + ⁣/*^mev^* mice after renal IRI (*n* = 3 in each group). **(D)** H&E staining of wild-type and *Ptpn6*^+/mev^ mice after renal IRI; the quantitative results are shown. Scale bars = 50 μm. The pathological scoring is as follows: score 1, less than 10%; score 2, 10% to less than 25%; score 3, 25–75%; and score 4, more than 75% of cortex with tubular damage. **(E)** TUNEL in the wild-type and *Ptpn6*^+/mev^ mouse groups after renal I/R and their quantitative results. Scale bars = 50 μm. **(F)** Accumulation of ROS levels in the kidneys of ROS in the wild-type and *Ptpn6*^+/mev^ mouse groups after renal I/R. Scale bars = 50 μm. **(G,H)** Quantitative results of H&E staining and TUNEL staining. *p < 0.05 and **p < 0.01.

A mouse renal IRI model was established with three 7-week-old *Ptpn6*^+/mev^ mice and three wild-type littermates. The steps for I/R model establishment are shown in Figure 1A, as described previously (14). Before surgery, the mice were anesthetized by intraperitoneal injection with 100 mg/kg pentobarbital sodium. Each mouse was placed on a temperature-controlled heating blanket and fitted with a rectal thermometer probe connected to a thermal feedback controller (RWD Life Science, China), and the rectal temperature was maintained at 37 ± 0.5°C. An abdominal midline incision was made, and then the right kidney of the mice was resected. The pedicle of the left kidney was clamped for 34 min with a non-traumatic vascular clamp before intraperitoneal injection of heparin (50 U/kg). During the clamping time, the abdominal midline incision was covered with a surgical dressing to keep the kidney warm and hydrated. The sham controls underwent similar procedures except for left kidney pedicle clamping. At 24 h post-IRI, the mice were sacrificed, and the left kidneys were removed for histologic and RNA analyses. For fenofibrate intervention, three mice were i.p. injected with 100 mg/kg/d fenofibrate (Sigma-Aldrich, Germany) and three mice were i.p. injected with corn oil (MedChemExpress, United States) from day 0 to day 20 before IRI surgery.

### Histomorphological Examination

Left renal kidney specimens were processed and sectioned as reported previously (15). Then, the sections were stained with hematoxylin-eosin (H and E) for histomorphological examination. A semiquantitative method was used to score the percentage of tubular damage according to the following metric: score 1, less than 10%; score 2, 10% to less than 25%; score 3, 25% to 75%; score 4, higher than 75% of cortex with tubular damage. At least three fields per section were analyzed by two different pathologists.

### Western Blot Analysis

For western blot analysis, proteins were extracted from mouse kidney tissues and processed according to a routine procedure. The antibodies used in this experiment were SHP-1 (E1U6R) rabbit monoclonal antibody and β-actin (13E5) rabbit monoclonal antibody (Cell Signaling Technology, United States), which were diluted as 1:1000. Images were obtained with a DM IL LED microscope (Leica Microsystems, Germany).

### Terminal Deoxynucleotidyl Transferase (TdT)-Mediated dUTP Nick-End Labeling Staining Assay

Apoptosis was confirmed with a terminal deoxynucleotidyl transferase (TdT)-mediated dUTP nick-end labeling (TUNEL) staining assay. TUNEL staining was conducted according to the kit manufacturer’s instructions (Beyotime, China).

### RNA Isolation and Deep Sequencing

Total RNA samples from the left kidney tissues were isolated with TRIzol reagent (Thermo Fisher Scientific, United States). An Agilent 2100 Bioanalyzer was used to characterize the quality of the *in vitro* RNA transcripts. The RNA integrity numbers (RINs) of all samples were greater than 8.0.

The poly-A-containing mRNA molecules were purified with poly-T oligo-attached magnetic beads using two rounds of purification. A SuperScript Double-Stranded cDNA Synthesis Kit (Thermo Fisher Scientific, United States) was used to synthesize the double-stranded cDNA. Further library preparation was performed using a TruSeq™ RNA Sample Preparation Kit (cat# FC-122-1001, Illumina, United States). The libraries were sequenced as 2 × 150 bp single reads using an Illumina HiSeq 2000 according to the manufacturer’s instructions. We removed adaptor, low-complexity, and low-quality sequences from the raw reads. The remaining clean reads were used for further analyses.

### Gene Expression Analysis

TopHat v2.1.0 was used with the default parameters to generate acceptable alignments for Cufflinks, which was used to align the RNA sequencing (RNA-seq) paired-end reads against the reference genome, Ensembl release 90 GRCm38.p5 (16). The expression of the annotated genes in the RNA-seq data was evaluated in fragments per kilobase million (FPKM) using Cufflinks. The following formula was used to calculate the FPKM value: FPKM = (number of mapped fragments) × 10^3^ × 10^6^/[(length of transcript) × (total number of fragments)]. Log transformation and zero-mean normalization were used to normalize the expression data for comparisons. The false discovery rate (FDR) of <0.05, after applying Benjamini-Hochberg correction, was chosen for determining significant DEGs.

An online analysis tool, the Protein ANalysis THrough Evolutionary Relationships (PANTHER) classification system, was used to annotate the DEGs into three major Gene Ontology (GO) domains: the molecular function, biological process, and cellular component domains (17). We also used the STRING system to analyze the most significantly (FDR < 0.005) enriched Kyoto Encyclopedia of Genes and Genomes (KEGG) pathways in DEGs (18, 19). STRING is an online analysis tool that can provide customized protein-protein networks and functional characterization of user-uploaded gene/measurement sets. The results of enrichment analysis were presented after Bonferroni correction for multiple comparisons.

### Validation of Differentially Expressed Genes

Reverse Transcription-Polymerase Chain Reaction (RT-PCR) was carried out with a LightCycler^®^ 480 II real-time RT-PCR system (Roche, Switzerland). RNA samples from the kidneys of 3 knockdown mice and 3 wild-type littermates after renal IRI were used in reverse transcription reactions. cDNA was synthesized using an oligo-dT reverse primer and a PrimeScript™ RT reagent kit (Takara, Japan). The primers used for RT-PCR validation of genes are listed in Supplementary Table 1. The expression of the GAPDH housekeeping gene was used to normalize the data. Gene expression was quantified with the 2^–ΔΔCT^ method, and the results are expressed as fold change (FC) relative to the levels in the corresponding control samples (20).

### Reactive Oxygen Species Staining Assay

Detection of ROS was performed on frozen sections using dihydroethidium (D7008, Sigma-Aldrich, United States) which was diluted as 1:500 for 30 min at 37°C in a dark incubator. DAPI (4′, 6-diamidino-2-phenylindole, Beyotime, China) was added after the slides were dry and incubated for 10 min in the dark at room temperature. Slides were washed three times for 5 min each in phosphate buffered saline (pH 7.4) on a shaker. After the plate was blocked with an antifade mounting medium (Servicebio, China), the images were observed and acquired under a fluorescence microscope.

### Statistics

GraphPad Prism 8.0 (GraphPad Software Inc., United States) was used to perform statistical analyses. All data are presented as the mean ± standard deviation. We performed Student’s unpaired *t*-tests for comparisons between two groups. All experiments were repeated more than three times. *P* < 0.05 was considered statistically significant.

## Results

### Development of Renal Renal Ischemia-Reperfusion Injury in Ptpn6 ^+/mev^ Mice

First, we validated the mutation and protein expression of SHP-1 in *Ptpn6*^+/mev^ mice by Sanger sequencing and western blotting, respectively (Supplementary Figures 1A–C). According to the process shown in Figure 1A, we then established IRI models in the wild-type ^+/+^ and *Ptpn6*^+/mev^ groups and performed RNA sequencing and data analysis. Mice in the *Ptpn6*^+/mev^ group exhibited more severe injury than those in the wild-type^+/+^ group, with higher serum creatinine (CR) and urea nitrogen (BUN) levels (Figures 1B,C). H and E staining revealed that tubular injury was significantly worse in the *Ptpn6*^+/mev^ group than in the wild-type group (Figure 1D). The TUNEL-positive areas of the kidneys were more intensely stained in *Ptpn6*^+/mev^ mice than in wild-type mice, and the positive areas were larger (Figure 1E). Quantitative results of H&E staining and TUNEL staining were also shown in Figures 1G–H. ROS staining results showed that *Ptpn6* ^+/mev^ mice produced more ROS than wild-type mice after IRI (Figure 1F). This finding suggests that the increased apoptosis of TECs in mice with SHP-1 deficiency contributes to renal IRI. Together, these results indicate that SHP-1 might be involved in apoptosis regulation and may exert a protective effect against renal IRI.

### Differentially Expressed Genes in Kidney Samples From Mice After Renal Ischemia-Reperfusion Injury

We extracted RNA from the kidneys of three C57BL/6J-*Ptpn6*^+/mev^ mice and three C57BL/6J control mice after renal IRI. Samples from individual mice were sequenced for bioinformatics analysis. The ischemia/reperfusion time was fixed at 34 min/24 h. After removing reads that could be mapped to ribosomes, a total of 152.67 million mRNA sequencing (mRNA-seq) reads were obtained. An average of 23.9 million reads of mRNA-seq data were generated per mRNA sample with a read length of 150 bp (paired-end reads) and an expected insertion size of 200 bp. The alignment rate against the reference genome (see section “Materials and Methods”) ranged from 94.07% (sample WT-3) to 92.00% (sample WT-2), with average mapping rates of 93.30 and 93.72% for the control and *Ptpn6*^+/mev^ samples, respectively (Supplementary Table 2).

**FIGURE 2:**
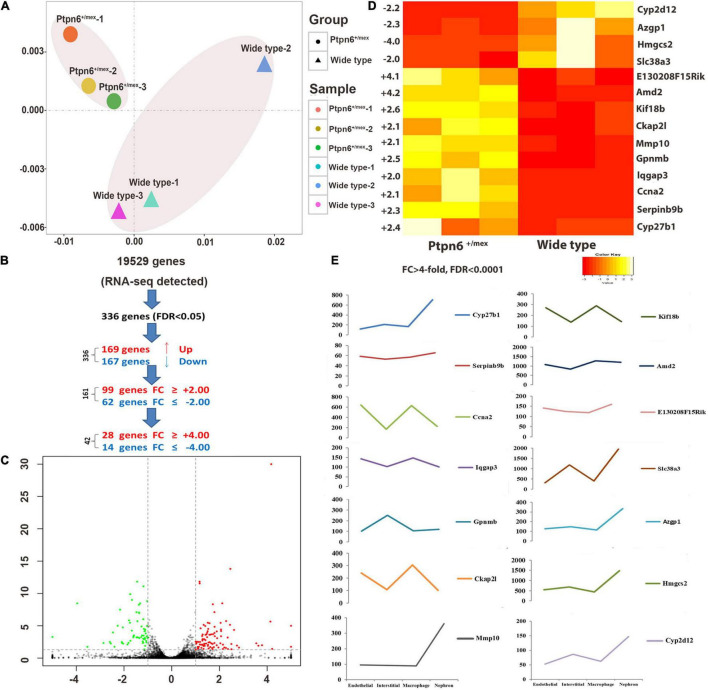
Differentially expressed genes (DEGs) in the kidneys of wild-type and *Ptpn6*^+/mev^ mice after renal IRI. **(A)** The PCA plot of the *Ptpn6*^+/mev^ samples (circles) shows distinct differences between samples from wild-type mice (triangles): each graph represents all DEGs of one animal, and the most similar *Ptpn6*^+/mev^ samples (circles) are located the closest. **(B)** A total of 336 genes out of 19,529 genes detected by RNA-seq were differentially expressed (FDR < 0.05). **(C)** A total of 161 genes showed at least twofold up-or down-regulation at FDR < 0.05. **(D)** Fourteen highly DEGs (FDR < 0.0001) in homozygous *Ptpn6*^+/mev^ kidneys compared to wild-type littermates. **(E)** Expression changes of 14 DEGs in different types of renal cells.

As presented in the principal component analysis (PCA) plot, after IRI, the kidneys of three *Ptpn6* ^+/mev^ mice and control mice showed differences in overall gene expression (Figure 2A). A total of 336 genes were differentially expressed in the kidney samples (FDR < 0.05). Of the 336 DEGs, 169 were upregulated, and 167 were downregulated in *Ptpn6*^+/mev^ mice after IRI. A total of 161 genes showed | FC| > 2 with FDR < 0.05, of which 42 genes with | FC| > 4 (Figure 2B). Of the 161 genes significantly changed after IRI, 99 were upregulated, and 62 were downregulated in *Ptpn6*^+/mev^ mice (Figure 2C). These 161 genes were DEGs in the following analysis (Supplementary Table 3).

The FDR and the largest | FC| were used to filter the gene list. Fourteen genes were differentially expressed (FDR < 0.0001) with | FC| > 4 in the kidneys of *Ptpn6*^+/mev^ mice (Figure 2D). Different types of cells are present in the kidney, and distinct translational signatures were identified in the nephron, interstitial cell populations, vascular endothelium, and macrophages in mice (21). By analyzing the Gene Expression Omnibus (GEO) dataset (GSE52004), we found that in the 11 highly overexpressed genes in *Ptpn6*^+/mev^ mice after IRI, *Cyp27b1*, *Mmp10*, *Hmgcs2*, *Azgp1*, *Cyp2d12*, and *Slc38a3* were expressed mostly in nephrons, and *Ckap2l* was expressed mostly in macrophages. The genes *Ccna2*, *Kif18b*, and *Lqgap3* were expressed in both the endothelium and macrophages. *Gpnmb* was expressed mostly in interstitial cells (Figure 2E).

### Gene Ontology Analysis and Kyoto Encyclopedia of Genes and Genomes Pathway Analysis of Mouse Kidney Samples After Renal Ischemia-Reperfusion Injury

The list of DEGs was uploaded to the online PANTHER version 16 classification system, which annotated the genes into three major GO domains: the molecular function, biological process, and cellular component domains (17). Among 161 DEGs, 104 genes were annotated to molecular functions. The PANTHER classification system annotated 49 genes (47.1%) to the catalytic activity category (GO:0003824) and 33 genes (31.7%) to the binding category (GO:0005488) in the molecular function domain. The most enriched terms in these two categories were the hydrolase activity (GO:0016787) and protein binding (GO:0005515) terms, respectively (Supplementary Figure 2A). Moreover, DEGs were also enriched in molecular function categories such as the molecular function regulator (GO:0098772) and transporter activity (GO:0005215) categories. In the biological process domain, a total of 66 genes were annotated to the cellular process category (GO:0009987), and 44 genes were annotated to the metabolic process category (GO:0008152) (Supplementary Figure 2B). The two most represented terms in the cellular process category were the cellular component organization (GO:0016043) and cell cycle (GO:0007049) terms. The organic substance metabolic process (GO:0071704) and primary metabolic process (GO:0044238) terms were the two most enriched terms in the metabolic process category. In the cellular component domain, 45 genes were annotated to the organelle category (GO:0043226), 33.3% of which were related to the cytoskeleton (GO:0005856), and 64 genes were annotated to the cell category (GO:0005623), 58.3% of which were related to the intracellular space (GO:0005622) (Supplementary Figure 2C). The PANTHER classification system (protein classes) sorted 33 genes to the metabolite interconversion enzyme (PC00262), 18 genes to oxidoreductase (PC00176) and 12 genes to cytoskeletal protein (PC00085) (Supplementary Figure 2D). Additional information on all gene annotations and protein classifications is available upon further request.

**FIGURE 3:**
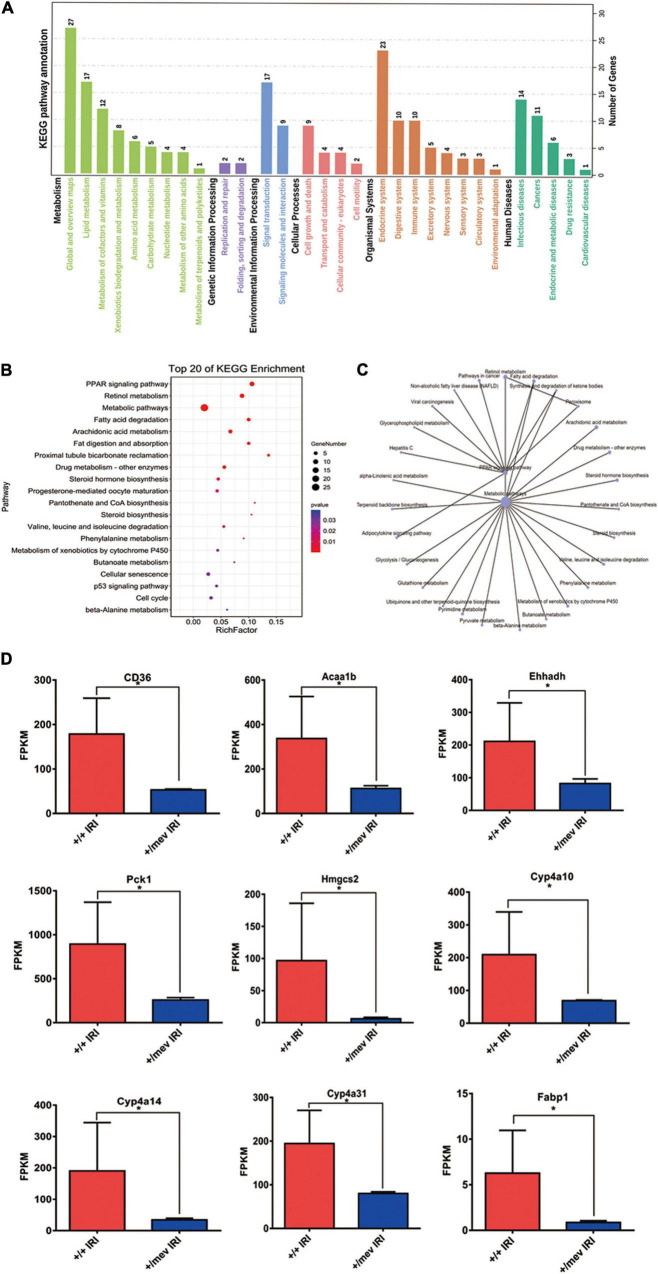
Kyoto encyclopedia of genes and genomes (KEGG) analysis of all DEGs and the expression of PPARα signaling pathway related genes. **(A)** KEGG pathway annotation of all DEGs. **(B)** The top 20 enriched KEGG pathways of all DEGs. A larger rich factor indicates a higher degree of enrichment. The plot is plotted with the pathways ranked by *P*-value from smallest to largest for the top 20. **(C)** KEGG enrichment network diagram of all DEGs. **(D)** The expression of PPARα signaling pathway related genes were obtained by RNA-seq analysis. *p < 0.05.

We also performed enrichment analysis of KEGG pathways in DEGs using the online STRING classification system (18, 19). The most significantly enriched (FDR < 0.005) KEGG pathways for the 62 genes that were significantly down-regulated more than twofold were the peroxisome-proliferator-activated receptor alpha (PPARα) signaling pathway and retinol metabolism (Figures 3A–C). In these two pathways, we identified the nine PPARα signaling pathway-related genes *CD36*, *Fabp1*, *Acaa1b*, *Ehhadh*, *Cyp4a10*, *Cyp4a14*, *Cyp4a31*, *Pck1*, and *Hmgcs2*. Among these genes, *CD36* and *Fabp1* are related to fat absorption, while *Acaa1b*, *Ehhadh*, *Cyp4a10*, and *Cyp4a14* are related to fatty acid degradation. The expression of genes encoding *Ptpn6*-interacting proteins in kidney samples from *Ptpn6*^+/mev^ mice after IRI is shown in Figure 3D. Then we found that the expression of *Ppara* was also significantly decreased in Ptpn6^+/mev^ mice than in wild-type mice after IRI (FC −1.63, FDR = 0.003). We validated the expression of the nine genes involved in the PPARα signaling pathway in the *Ptpn6*^+/mev^ mice and wild-type mice after IRI by RT-PCR. We also compared the expression of these genes in the control kidneys without IRI in Ptpn6 ^+/mev^ and wild-type mice and found that there were no significant changes (| FC| < 1.5 with FDR > 0.05) (Supplementary Table 4). Moreover, for the 99 significantly upregulated genes in *Ptpn6*^+/mev^ mice after IRI, the most significantly enriched KEGG pathways were the mitotic cell cycle process and cell division pathways. Previous studies have revealed that cell cycle function-associated genes are generally upregulated at the peak of injury (24 h after IRI) (22). In this study, key kinases involved in the proper spindle and kinetochore assemblies, such as *Aurkb*, *Plk1*, *Cdk1*, *Tpx2*, and *Cdca3*, were upregulated in the *Ptpn6*^+/mev^ group compared with the wild-type^+/+^ group at 24 h after IRI.

Src homology region 2 domain-containing phosphatase-1 has two SH2 binding domains and one PTP catalytic domain. SHP-1 inhibits signal transduction from receptors in many cell types^1^ through interaction with the immunoreceptor tyrosine-based inhibitory motif (ITIM) (23–25). After IRI, we found five upregulated and nine downregulated genes in *Ptpn6*^+/mev^ mice that encoded proteins with ITIMs (FDR < 0.05), of which three genes were upregulated and five genes were downregulated by more than twofold (Supplementary Table 5). Of these eight genes, six genes were related to lipid metabolism. In the kidneys, proximal TECs use fatty acids as their main energy source due to the high energy demand. A previous study indicated that overexpression of ATF6a transcriptionally downregulated PPARα, leading to reduced fatty acid β-oxidation, enhanced apoptosis and reduced cell viability in a human proximal TEC line (HK-2) (26). Since apoptosis in TECs was also enhanced in our study, we suggest that the PPARα signaling pathway plays an important role in the development of renal IRI in SHP-1-knockdown mice.

### Peroxisome-Proliferator-Activated Receptor Alpha Agonists Attenuate Renal Ischemia-Reperfusion Injury in Ptpn6^+/mev^ Mice

**FIGURE 4:**
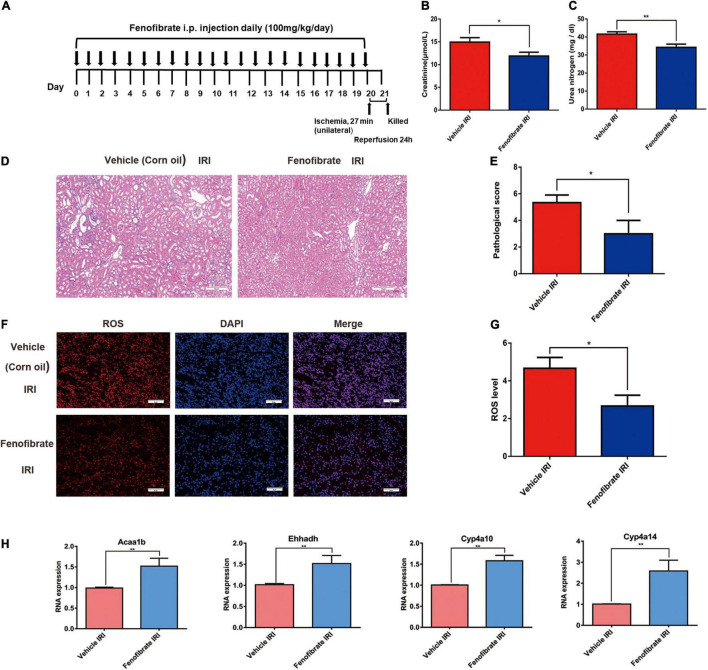
Fenofibrate reverses renal tubular injury caused by a decrease in lipid metabolism gene expression and the accumulation of reactive oxygen species mediated by SHP-1 insufficiency in mice with ischemia-reperfusion injury. **(A)** Schematic diagrams of the experimental procedure. **(B,C)** Levels of creatinine and urea nitrogen in *Ptpn6*^+/mev^ mice given fenofibrate and vehicle control (corn oil). **(D,E)** H and E staining in the kidneys of Ptpn6^+/mev^ mice given fenofibrate and in those given vehicle control after IRI; the quantitative results are shown. Scale bars = 50 μm. **(F,G)** Accumulation of ROS levels in the kidneys of Ptpn6^+/mev^ mice given fenofibrate and in those given vehicle after IRI; the quantitative results are shown. Scale bars = 50 μm. **(H)** Expression of *Acaa1b*, *Ehhadh*, *Cyp4a10*, and *Cyp4a14* in *Ptpn6*^+/mev^ mice given fenofibrate and in those given vehicle control after IRI. *p < 0.05, **p < 0.01.

**FIGURE 5:**
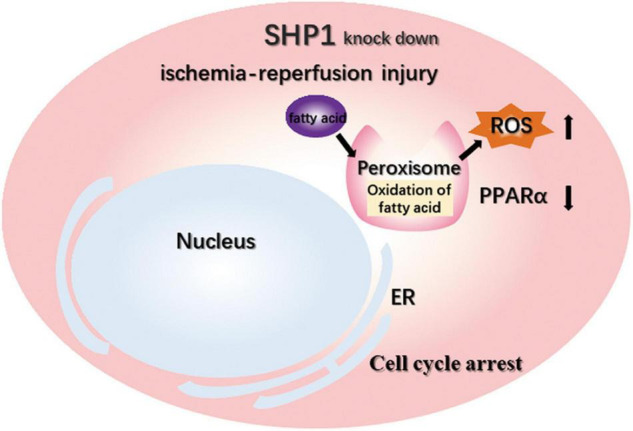
Schematic of the mechanisms that aggravate IRI after SHP-1 knockdown, possibly through downregulation of the PPARα signaling pathway.

To confirm that PPARα signaling plays a central role in the aggravation of renal IRI induced by SHP-1 insufficiency, we activated PPARα signaling by administrating the agonist fenofibrate or vehicle control (corn oil) to six Ptpn6^+/mev^ mice for 3 weeks to activate the PPARα signaling pathway, followed by IRI (Figure 4A). When the mice were killed 24 h after IRI, the serum CR and BUN of three mice taking fenofibrate were significantly lower than those of three mice taking corn oil (Figures 4B,C). Furthermore, as assessed using H and E staining and ROS staining analysis, the kidneys of mice in the fenofibrate-treated group showed attenuation of renal injury and reduction of ROS levels (Figures 4D–G). We extracted RNA from the kidney after IRI for RT-PCR to confirm that the expression of *Acaa1b*, *Ehhadh*, *Cyp4a10*, and *Cyp4a14*, which are related to fatty acid degradation, was significantly increased in mice treated with fenofibrate but not corn oil (Figure 4H). Taken together, these *in vivo* results suggested that SHP-1 knockdown may lead to the downregulation of fatty acid oxidation, along with an increase in ROS production and renal tubular damage. This phenotypic combination ultimately contributes to extracellular matrix production and promotes mesangial matrix or basement membrane thickening after IRI in *Ptpn6*^+/mev^ mice. A schematic of SHP-1 insufficiency leading to the development of renal IRI via suppression of PPARα signaling is shown in Figure 5.

## Discussion

Src homology region 2 domain-containing phosphatase-1, a highly conserved intracellular PTP, is expressed primarily in hematopoietic cells and plays a critical role in deciding the fate of immune cells by modulating the duration and amplitude of a downstream cascade transduced via receptors in both mice and humans (27). In several studies, SHP-1 has been considered a negative regulator of hemopoietic and immune cytokine signaling (28, 29). However, the biological function of SHP-1 in epithelial cells is not well understood. Inconsistent with these findings, a previous study conducted in our laboratory demonstrated that SHP-1 can bind to and dephosphorylate ASK1 to inhibit its activation, thus repressing apoptosis in TECs in SHP-1 knockout mice after renal IRI, which is a new insight into the biological function of SHP-1 in epithelial cells (12).

Peroxisome-proliferator-activated receptor alpha, which is expressed primarily in fat, liver, heart, muscle, renal cortex, and other tissues with high catabolic rates, is the main regulator of lipid and energy metabolism (30). PPARα also participates in mediating inflammation and apoptosis caused by injury (31). Previous studies have shown that PPARα plays a protective role against IRI in many solid organs. For example, PPARα activation can enhance antioxidation and the anti-inflammatory response in the context of hepatic IRI by increasing the expression of antioxidant enzymes and inhibiting the activity of NFκB (32). In myocardial ischemia-reperfusion, branched-chain amino acids can render the heart vulnerable to IRI by enhancing GCN2/ATF6/PPARα pathway-dependent fatty acid oxidation and metabolism (33). Similarly, ginsenoside Rb3 can activate the PPARα pathway, thus protecting against myocardial IRI. Other studies have also suggested that PPARα activation can protect against myocardial IRI in type 2 diabetic rats through PI3K/Akt and NO pathway activation (34). In the kidneys, PPARα expression decreases after renal IRI. Agonists of PPARα have been found to ameliorate renal IRI in mice and rat models (26, 35). Moreover, PPARα can decrease kidney fibrosis development in TECs by regulating fatty acid oxidation (36). Decreased expression of PPARα after renal IRI can promote fibrosis, and overexpression of PPARα induced by miR-21 deletion can prevent ureteral obstruction-induced injury and fibrosis of the kidneys (37).

In this study, the PPARα signaling pathway was the most significantly enriched KEGG pathway for the downregulated genes in SHP-1-knockdown mice compared with wild-type mice after IRI. The correlation between SHP-1 and the PPARα signaling pathway has not been reported previously. DEGs in porcine intramuscular adipocytes differentiated with exogenous TNF-α and serotonin, including *Ptpn6*, are enriched in the PPAR signaling pathway (38). In our study, nine downregulated genes (*CD36*, *Acaa1b*, *Ehhadh*, *Pck1*, *Hmgcs2*, *Cyp4a10*, *Cyp4a31*, *Cyp4a14*, and *Fabp1*) in the PPARα signaling pathway were also found to be related to fatty acid degradation and absorption, which results in the accumulation of triglycerides in injured TECs (39). Furthermore, it has been reported that *Ehhadh* can be upregulated by a new selective PPARα agonist, CP775146, which prevents lipid accumulation in obese mice (40). *Pck1*, *Bcl-2*, and PPARα levels can be increased by miR-292-5p downregulation to protect against myocardial IRI (41). Gene expression profiling has shown that *Fabp1* and *Hmgcs2* are upregulated in hepatocyte humanized mice treated with the PPARα agonist fenofibrate (42). In summary, PPARα expression decreases as peroxisomal fatty acid oxidation is inhibited in the context of renal IRI. These changes are consistent with the findings of previous studies and might be leading mechanisms by which SHP-1 insufficiency in the kidneys can exacerbate injury and apoptosis of TECs after renal IRI (35, 43).

Reactive oxygen species is excessively generated after IRI, which causes severe damage inside tissues transplantable tissues such as the heart, liver, and kidney (44). There are several studies suggesting that the generation of ROS during IRI occurs by a well-defined mechanism (45). SHP-1 suppression is related to the development of airway inflammation and increased ROS levels in airway epithelia under conditions of oxidative stress (46). Suppression of the SHP-1 function promotes a further increase in the intracellular ROS level by eliciting amplified and prolonged activation of endogenous ROS (11). In this study, insufficiency of SHP-1 lead to the suppression of the PPARα signaling pathway after IRI, while the activation of the PPARα signaling pathway plays a protective role in reducing ROS in the heart, liver and kidney (47–49).

Since SHP-1 is involved in various signaling pathways, we used next-generation sequencing to characterize the DEGs in the kidneys of SHP-1-knockdown mice after renal IRI. The RNA-seq studies enabled complicated analyses of all biological pathways in one experiment, which was important for profiling the characteristic gene changes after IRI. We were able to identify several essential pathways influenced by SHP-1, such as the PPARα signaling pathway and the mitosis pathway. A major limitation of this study is that the number of animals used in the experiment is relatively low, while the changes between different groups, although significant, are still small. We hope to expand the number of samples in future research. In summary, our results show that insufficiency of SHP-1 inhibits the expression of genes in the PPARα signaling pathway, especially those which were related to fatty acid degradation, thereby leading to increasing in ROS and aggravating IRI. We also demonstrated that the changes in gene expression were due to SHP-1 deficiency-induced different responses to IRI rather than deficiency of SHP-1. It will be interesting to analyze the fatty acid profiles in the kidneys of the SHP-1 insufficiency mice and the wild-type mice after IRI. Our findings help to demonstrate the mechanisms by which SHP-1 insufficiency could lead to the development of renal IRI and provide new insights into the roles of fatty acid degradation and mitosis in renal IRI.

## Data Availability Statement

The data presented in the study are deposited in the GEO repository, accession number GSE200717.

## Ethics Statement

The animal study was reviewed and approved by the Institutional Research Ethics Committee of Navy Medical University, Shanghai, China. Written informed consent was obtained from the owners for the participation of their animals in this study.

## Author Contributions

LZ and XD designed the studies and supervised the project. SY and MS performed most of the experiments and co-wrote the manuscript. JF and JC performed to establish the mice model construction. YL and JC performed the histological analysis. SY performed the bioinformatics analysis. MS analyzed the data and provided statistical guidance. HT performed some of the RT-PCR experiments and provided experimental guidance. All authors contributed to the article and approved the submitted version.

## Conflict of Interest

The authors declare that the research was conducted in the absence of any commercial or financial relationships that could be construed as a potential conflict of interest.

## Publisher’s Note

All claims expressed in this article are solely those of the authors and do not necessarily represent those of their affiliated organizations, or those of the publisher, the editors and the reviewers. Any product that may be evaluated in this article, or claim that may be made by its manufacturer, is not guaranteed or endorsed by the publisher.

## Footnotes

1 http://biogps.org and www.immgen.org

## References

[1] Perico N Cattaneo D Sayegh MH Remuzzi G. Delayed graft function in kidney transplantation. *Lancet.* (2004) 364:1814–27. 10.1016/S0140-6736(04)17406-0 15541456

[2] Keilhack H Hellman U van Hengel J van Roy F Godovac-Zimmermann J Böhmer FD. The protein-tyrosine phosphatase SHP-1 binds to and dephosphorylates p120 catenin. *J Biol Chem.* (2000) 275:26376–84. 10.1074/jbc.m001315200 10835420

[3] Štefanová I Hemmer B Vergelli M Martin R Biddison WE Germain RN. TCR ligand discrimination is enforced by competing ERK positive and SHP-1 negative feedback pathways. *Nat Immunol.* (2003) 4:248–54. 10.1038/ni895 12577055

[4] Blanchette J Racette N Faure R Siminovitch KA Olivier M. Leishmania-induced increases in activation of macrophage SHP-1 tyrosine phosphatase are associated with impaired IFN-γ-triggered JAK2 activation. *Eur J Immunol.* (1999) 29:3737–44. 10.1002/(SICI)1521-4141(199911)29:113.0.CO;2-S 10556830

[5] Yousefi S Simon H-U. SHP-1: a regulator of neutrophil apoptosis. *Semin Immunol.* (2003) 15:195–9. 10.1016/S1044-5323(03)00033-2 14563118

[6] Sulub Y DeRudder J. Determination of polymer blends composed of polycarbonate and rubber entities using near-infrared (NIR) spectroscopy and multivariate calibration. *Polym Test.* (2013) 32:802–9. 10.1016/j.polymertesting.2013.03.008

[7] Thangaraju M Sharma K Liu D Shen SH Srikant CB. Interdependent regulation of intracellular acidification and SHP-1 in apoptosis. *Cancer Res.* (1999) 59:1649–54. 10197642

[8] An H Hou J Zhou J Zhao W Xu H Zheng Y Phosphatase SHP-1 promotes TLR- and RIG-I-activated production of type I interferon by inhibiting the kinase IRAK1. *Nat Immunol.* (2008) 9:542–50. 10.1038/ni.1604 18391954

[9] Sundaresan M Yu Z-X Ferrans VJ Irani K Finkel TJS. Requirement for generation of H(2)O(2) for platelet-derived growth factor signal tran sduction. *Science.* (1995) 270:296–9. 10.1126/science.270.5234.296 7569979

[10] Meng TC Fukada T Tonks NK. Reversible oxidation and inactivation of protein tyrosine phosphatases in vivo. *Mol Cell.* (2002) 9:387–99. 10.1016/S1097-2765(02)00445-8 11864611

[11] Krötz F Engelbrecht B Buerkle MA Bassermann F Bridell H Gloe T The tyrosine phosphatase, SHP-1, is a negative regulator of endothelial superoxide formation. *J Am Coll Cardioly.* (2005) 45:1700–6. 10.1016/j.jacc.2005.02.039 15893190

[12] Tian H Tan R Ye B Yan S Sui M Zhao W SHP-1 inhibits renal ischemia reperfusion injury via dephosphorylating ASK1 and suppressing apoptosis. *Biochem Bioph Res Commun.* (2019) 513:360–7. 10.1016/j.bbrc.2019.03.187 30961932

[13] Kamata T Yamashita M Kimura M Murata K Inami M Shimizu C. SRC homology 2 domain-containing tyrosine phosphatase SHP-1 controls the development of allergic airway inflammation. *J Clin Invest.* (2003) 111:109–19. 10.1172/JCI15719 12511594 PMC151831

[14] Zhang S Han C-H Chen X-S Zhang M Xu L-M Zhang J-J Transient ureteral obstruction prevents against kidney ischemia/reperfusion injury via hypoxia-inducible factor (HIF)-2α activation. *PLoS One.* (2012) 7:e29876. 10.1371/journal.pone.0029876 22295069 PMC3266244

[15] Gholampour H Moezi L. Shafaroodi H. Aripiprazole prevents renal ischemia/reperfusion injury in rats, probably through nitric oxide involvement. *Eur J Pharmacol.* (2017) 813:17–23. 10.1016/j.ejphar.2017.07.032 28734929

[16] Trapnell C Roberts A Goff L Pertea G Kim D Kelley DR Differential gene and transcript expression analysis of RNA-seq experiments with TopHat and Cufflinks. *Nat Protoc.* (2012) 7:562–78. 10.1038/nprot.2012.016 22383036 PMC3334321

[17] Mi H Ebert D Muruganujan A Mills C Albou L-P Mushayamaha T PANTHER version 16: a revised family classification, tree-based classification tool, enhancer regions and extensive API. *Nucleic Acids Res.* (2021) 49:D394–403. 10.1093/nar/gkaa1106 33290554 PMC7778891

[18] Szklarczyk D Gable AL Nastou KC Lyon D Kirsch R Pyysalo S The STRING database in 2021: customizable protein–protein networks, and functional characterization of user-uploaded gene/measurement sets. *Nucleic Acids Res.* (2021) 49:D605–12. 10.1093/nar/gkaa1074 33237311 PMC7779004

[19] Kanehisa M Furumichi M Sato Y Ishiguro-Watanabe M Tanabe M. KEGG: integrating viruses and cellular organisms. *Nucleic Acids Res.* (2021) 49:D545–51. 10.1093/nar/gkaa970 33125081 PMC7779016

[20] Livak K Methods TSJ. Analysis of relative gene expression data using real-time quantitative PCR and the 2^–△△Ct^ Method. *Afr J Biotechnol.* (2012) 11:6226–33. 10.5897/AJB11.4117 11846609

[21] Liu J Krautzberger AM Sui SH Hofmann OM Chen Y Baetscher M Cell-specific translational profiling in acute kidney injury. *J Clin Invest.* (2014) 124:1242–54. 10.1172/JCI72126 24569379 PMC3938273

[22] Tae-Min K Victoria R Jonatan B-C Norma AB Peter JP Vishal SV Gene expression analysis reveals the cell cycle and kinetochore genes participating in ischemia reperfusion injury and early development in kidney. *PLoS One.* (2011) 6:e25679. 10.1371/journal.pone.0025679 21980527 PMC3181346

[23] Daëron M Jaeger S Du Pasquier L Vivier E. Immunoreceptor tyrosine-based inhibition motifs: a quest in the past and future. *Immunol Rev.* (2008) 224:11–43. 10.1111/j.1600-065x.2008.00666.x 18759918

[24] Staub E Rosenthal A Hinzmann B. Systematic identification of immunoreceptor tyrosine-based inhibitory motifs in the human proteome. *Cell Signal.* (2004) 16:435–56. 10.1016/j.cellsig.2003.08.013 14709333

[25] Zhang J Somani A-K Siminovitch KA. Roles of the SHP-1 tyrosine phosphatase in the negative regulation of cell signalling. *Semin Immunol.* (2000) 12:361–78. 10.1006/smim.2000.0223 10995583

[26] Jao T-M Nangaku M Wu C-H Sugahara M Saito H Maekawa H ATF6α downregulation of PPARα promotes lipotoxicity-induced tubulointerstitial fibrosis. *Kidney Int.* (2019) 95:577–89. 10.1016/j.kint.2018.09.023 30639234

[27] Dong Q Siminovitch KA Fialkow L Fukushima T Downey GP. Negative regulation of myeloid cell proliferation and function by the SH2 domain-containing tyrosine phosphatase-1. *J Immunol.* (1999) 162:3220–30. 10.1100/tsw.2002.247 10092773

[28] Duchesne C Charland S Asselin C Nahmias C Rivard N. Negative regulation of β-catenin signaling by tyrosine phosphatase SHP-1 in intestinal epithelial cells. *J Biol Chem.* (2003) 278:14274–83. 10.1074/jbc.M300425200 12571228

[29] Speranza L Pesce M Patruno A Franceschelli S de Lutiis MA Grilli A Astaxanthin treatment reduced oxidative induced pro-inflammatory cytokines secretion in U937: SHP-1 as a novel biological target. *Mar Drugs.* (2012) 10:890–9. 10.3390/md10040890 22690149 PMC3366681

[30] Cheng CF Lian W-S Chen S-H Lai P-F Li H-F Lan Y-F Protective effects of adiponectin against renal ischemia-reperfusion injury via prostacyclin-PPARα-Heme oxygenase-1 signaling pathway. *J Cell Physiol.* (2012) 227:239–49. 10.1002/jcp.22726 21412771

[31] Maeda T Kishioka S. Chapter 13 PPAR and Pain. *Int Rev Neurobiol.* (2009) 85:165–77. 10.1016/S0074-7742(09)85013-7 19607969

[32] Gao Z Li Y-H. Antioxidant stress and anti-inflammation of PPARα on warm hepatic ischemia-reperfusion injury. *PPAR Res.* (2012) 2012:738785. 10.1155/2012/738785 23213319 PMC3503442

[33] Li Y Xiong Z Yan W Gao E Cheng H Wu G Branched chain amino acids exacerbate myocardial ischemia/reperfusion vulnerability via enhancing GCN2/ATF6/PPAR-α pathway-dependent fatty acid oxidation. *Theranostics.* (2020) 10:5623–40. 10.7150/thno.44836 32373236 PMC7196282

[34] Bulhak AA Jung C Östenson C Lundberg JO Sjöquist PO Pernow J. PPAR-α activation protects the type 2 diabetic myocardium against ischemia-reperfusion injury: involvement of the PI3-Kinase/Akt and NO pathway. *Am J Physiol Heart Circ Physiol.* (2009) 296:719–27. 10.1152/ajpheart.00394.2008 19151258

[35] Sivarajah A Chatterjee PK Hattori Y Brown PA Stewart KN Todorovic Z Agonists of peroxisome-proliferator activated receptor-alpha (clofibrate and WY14643) reduce renal ischemia/reperfusion injury in the rat. *Med Sci Monit.* (2002) 8:BR532–9. 10.1017/S1355770X11000337 12503032

[36] Kang HM Ahn SH Choi P Ko Y-A Han SH Chinga F Defective fatty acid oxidation in renal tubular epithelial cells has a key role in kidney fibrosis development. *Nat Med.* (2015) 21:37–46. 10.1038/nm.3762 25419705 PMC4444078

[37] Chau BN Xin C Hartner J Ren S Castano AP Linn G MicroRNA-21 promotes fibrosis of the kidney by silencing metabolic pathways. *Sci Transl Med.* (2012) 4:121ra18. 10.1126/scitranslmed.3003205 22344686 PMC3672221

[38] Tada A Islam MA Kober AH Fukuyama K Takagi M Igata M Transcriptome modifications in the porcine intramuscular adipocytes during differentiation and exogenous stimulation with TNF-α and serotonin. *Int J Mol Sci.* (2020) 21:638. 10.3390/ijms21020638 31963662 PMC7013444

[39] Johnson ACM Stahl A Zager RA. Triglyceride accumulation in injured renal tubular cells: alterations in both synthetic and catabolic pathways. *Kidney Int.* (2005) 67:2196–209. 10.1111/j.1523-1755.2005.00325.x 15882263

[40] Tang S Wu F Lin X Gui W Zheng F Li H. The effects of new selective PPARα agonist CP775146 on systematic lipid metabolism in obese mice and its potential mechanism. *J Diabetes Res.* (2020) 2020:4179852. 10.1155/2020/4179852 32455134 PMC7222497

[41] Zhu Z-D Ye J-Y Niu H Ma Y-M Fu X-M Xia Z-H Effects of microRNA-292-5p on myocardial ischemia-reperfusion injury through the peroxisome proliferator-activated receptor-α/-γ signaling pathway. *Gene Ther.* (2018) 25:234–48. 10.1038/s41434-018-0014-y 29670247

[42] de la Rosa Rodriguez MA Sugahara G Hooiveld GJEJ Ishida Y Tateno C Kersten S. The whole transcriptome effects of the PPARα agonist fenofibrate on livers of hepatocyte humanized mice. *BMC Genomics.* (2018) 19:443. 10.1186/s12864-018-4834-3 29879903 PMC5991453

[43] Gulati S Ainol Orak J Singh AK Singh I. Alterations of peroxisomal function in ischemia-reperfusion injury of rat kidney. *Biochim Biophys Acta.* (1993) 1182:291–8. 10.1016/0925-4439(93)90071-8 8399363

[44] Chouchani ET Pell VR Gaude E Aksentijević D Sundier SY Robb EL Ischaemic accumulation of succinate controls reperfusion injury through mitochondrial ROS. *Nature.* (2014) 515:431–5. 10.1038/nature13909 25383517 PMC4255242

[45] Chouchani Edward T Pell Victoria R James Andrew M Work Lorraine M Saeb-Parsy K Frezza C A unifying mechanism for mitochondrial superoxide production during ischemia-reperfusion injury. *Cell Metab.* (2016) 23:254–63. 10.1016/j.cmet.2015.12.009 26777689

[46] Jang MK Kim S-H Lee K-Y Kim T-B Moon KA Park CS The tyrosine phosphatase, SHP-1, is involved in bronchial mucin production during oxidative stress. *Biochem Bioph Res Commun.* (2010) 393:137–43. 10.1016/j.bbrc.2010.01.102 20117097

[47] Ibarra-Lara L Hong E Soria-Castro E Torres-Narváez JC Pérez-Severiano F Del Valle-Mondragón L Clofibrate PPARα activation reduces oxidative stress and improves ultrastructure and ventricular hemodynamics in no-flow myocardial ischemia. *J Cardiovasc Pharmacol.* (2012) 60:323–34. 10.1097/fjc.0b013e31826216ed 22691880

[48] Patterson AD Shah YM Matsubara T Krausz KW Gonzalez FJ. Peroxisome proliferator-activated receptor alpha induction of uncoupling protein 2 protects against acetaminophen-induced liver toxicity. *Hepatology.* (2012) 56:281–90. 10.1002/hep.25645 22318764 PMC3378765

[49] Hou X Shen YH Li C Wang F Zhang C Bu P PPARα agonist fenofibrate protects the kidney from hypertensive injury in spontaneously hypertensive rats via inhibition of oxidative stress and MAPK activity. *Biochem Bioph Res Commun.* (2010) 394:653–9. 10.1016/j.bbrc.2010.03.043 20226762

