# Supplementary material for: Transcriptomic Analysis Revealed an Important Role of Peroxisome-Proliferator-Activated Receptor Alpha Signaling in Src Homology Region 2 Domain-Containing Phosphatase-1 Insufficiency Leading to the Development of Renal Ischemia-Reperfusion Injury
Source: Front Med (Lausanne). 2022 May 10;9:847512. doi: 10.3389/fmed.2022.847512 (PMC9134314; doi:10.3389/fmed.2022.847512)
Supplement: Supplementary file 6 [file Data_Sheet_1.PDF]

Supplementary Material

1.1 Supplementary Figures

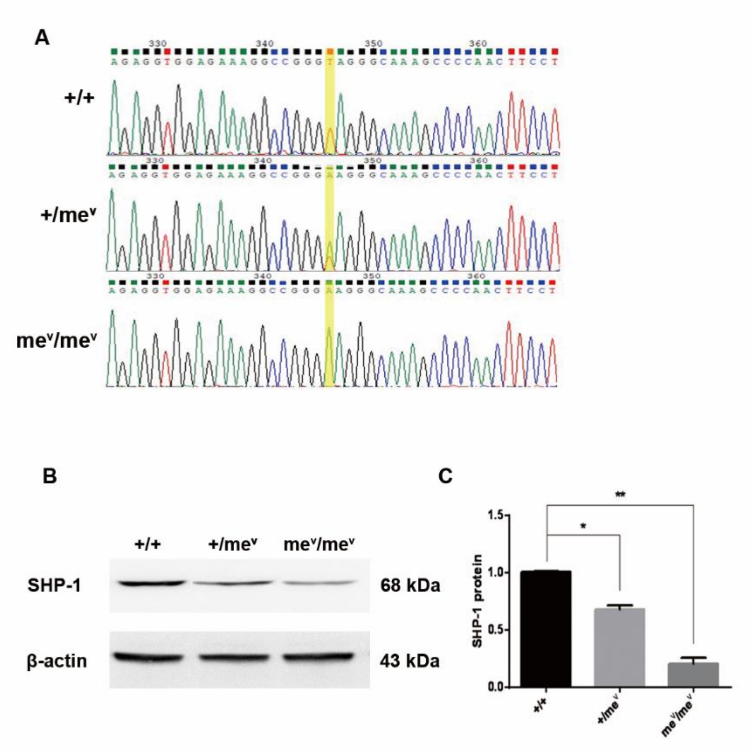

Supplementary Figure 1 (A) Shown is the mutation in Sanger sequencing result of wide type, Ptpn6$^{+/mev}$ and Ptpn6$^{mev /mev}$ mice. (B and C) Shown are the protein (B) and mRNA (C) expression level of SHP-1 in wide type , Ptpn6$^{+/mev}$ and Ptpn6$^{mev /mev}$ mice .

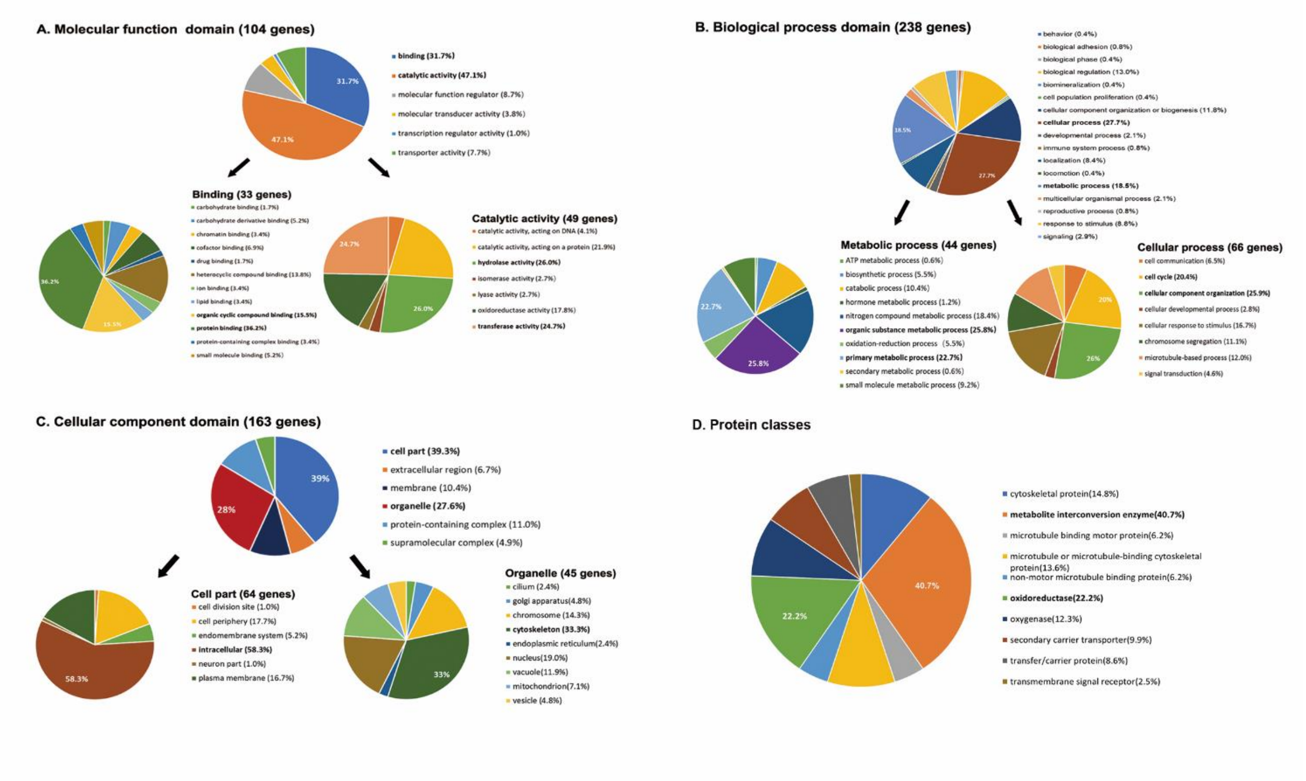

Supplementary Figure 2 The Gene Ontology (GO) annotation was based on (A) the molecular function, (B) biological processes, (C) cellular component domains and (D) protein classes of all DEPs (FDR<0.05, FCs >2).
